# Consumption of serum lipid-reducing drugs in Serbia compared with Scandinavian countries: a population-based study, 2004–2010

**DOI:** 10.1186/2050-6511-13-S1-A86

**Published:** 2012-09-17

**Authors:** Ana Sabo, Zdenko Tomić, Nebojša Stilinović, Boris Milijašević, Momir Mikov, Saša Vukmirović, Olga Horvat

**Affiliations:** 1 Department of Pharmacology, Toxicology and Clinical Pharmacology, Faculty of Medicine, University of Novi Sad, 21000 Novi Sad, Serbia

## Background

Serbia is one of the leading countries in the world with respect to mortality from cardiovascular diseases. Drugs that reduce serum lipids belong to the group of drugs that can significantly lower complications of cardiovascular diseases and their utilization in Serbia deserves special attention. The aim of this study was to measure the consumption of serum lipid reducing drugs in Serbia from 2004 to 2010, to compare these data with those from Scandinavian countries, and to compare the consumption of lipid-lowering drugs and the rate of mortality from cardiovascular diseases in these countries.

## Methods

A population-based study was undertaken to analyse lipid-lowering drug consumption using the Anatomical Therapeutic Chemical/Defined Daily Dose methodology. Cause-specific mortality rates were obtained from the WHOSIS annual report for the year 2009.

## Results

In 2010, a total of 966 DDD/1000 inh/day (DID) of all drugs, was used in Serbia, of which 38.9% belonged to drugs for cardiovascular diseases. While in Scandinavian countries 17.0–24.8% of drugs for cardiovascular diseases belonged to lipid-lowering drugs, in Serbia it was substantially lower (3%). In 2004 in Serbia, 1.50 DID of statins were used. In 2008, this value was 32.5 DID. In every investigated country, simvastatin made up more than 50% of the consumption of statins. After simvastatin, the next most frequently used statin was atorvastatin, with 5.52, 11.0, 11.2 and 24.8 DID, in Serbia, Denmark, Finland and Norway, respectively. In 2004 Serbia had the highest mortality rate for cardiovascular diseases among the investigated countries with 762/100.000 inhabitants and Norway has the lowest rate with 158/100.000 inhabitants.

## Conclusions

The use of lipid-lowering drugs is 6–8 times lower in Serbia as compared to Scandinavian countries but there is an evident rise in lipid lowering drugs consumption in Serbia during the investigated years.

